# Ergot of Rye in After-Pains

**Published:** 1847-05

**Authors:** 


*Ergot of Rye in After-Pains.*—Dr. Beatty observes that he has found the ergot most useful in preventing, or greatly moderating the severity of after-pains. He has seen a patient who was writhing from pain, which opium had not the power of controlling, speedily relieved by the ergot. He exhibits it for this purpose in the same manner as when his object is to restrain hemorrhage. He thinks that it acts by causing perfect contraction of the fibres of the uterus, and keeping them in this condition, and also by preventing the formation of clots in the interior of the uterus, by whose presence the spasmodic action is excited and kept up.—*Dub. Quart. Jour.—Ibid.*

